# Supplementary material for: Comparative effect of physical exercise versus statins on improving arterial stiffness in patients with high cardiometabolic risk: A network meta-analysis
Source: PLoS Med. 2021 Feb 16;18(2):e1003543. doi: 10.1371/journal.pmed.1003543 (PMC7924736; doi:10.1371/journal.pmed.1003543)
Supplement: S2 Table — (DOCX) [file pmed.1003543.s002.docx]

**S2 Table.** Search strategy for the MEDLINE database

| **Search set Medline** | **Search set Medline** |
| --- | --- |
| #1 type 2 diabetes [All Fields]  #2 T2DM [All Fields]  #3 chronic kidney disease [All Fields]  #4 CKD [All Fields]  #5 1 OR 2 OR 3 OR 4  #6 dyslipidemia [Mesh Terms]  #7 dyslipidemia [All Fields]  #8 6 OR 7  #9 hypertension [Mesh Terms]  #10 HTA [All Fields]  #11 9 OR 10  #12 hypercholesterolaemia [Mesh Terms]  #13 hypercholesterolemia [Mesh Terms]  #14 12 OR 13  #15 cardiovascular disease [All Fields]  #16 overweight [Mesh Terms]  #17 obesity [Mesh Terms]  #18 obese [All Fields]  #19 17 OR 18  #20 haemodialysis [All Fields]  #21 renal dialysis [Mesh Terms]  #22 hemodialysis [All Fields]  #22 renal [All Fields]  #23 dialysis  #24 22 AND 23  #25 20 OR 21 OR 22 OR 24  #26 metabolic syndrome [All Fields]  #27 5 OR 8 OR 11 OR 14 OR 15 OR 16 OR 19 OR 24 OR 25  #28 randomized controlled trial [All Fields]  #29 randomized trial [All Fields]  #30 randomized pilot study [All Fields]  #31 intervention [All Fields]  #32 28 OR 29 OR 30 OR 31  #33 Hydrosymethylglutary-coa reductase inhibitors [Mesh Terms]  #34 Hydrosymethylglutary-coa [All Fields]  #35 reductase [All Fields]  #36 inhibitors [All Fields]  #36 34 AND 35 AND 36 | #37 statin [All Fields]  #38 33 OR 36 OR 37  #39 atorvastatin [Mesh Terms]  #40 rosuvastatin calcium [Mesh Terms]  #41 rosuvastatin [All Fields]  #42 calcium [All Fields]  #43 41 AND 42  #44 rosuvastatin [All Fields]  #45 40 OR 43 OR 44  #46 fluvastatin [Mesh Terms]  #47 simvastatin [Mesh Terms]  #48 lovastatin [Mesh Terms]  #49 pitavastatin [All Fields]  #50 pravastatin [Mesh Terms]  #51 39 OR 45 OR 46 OR 47 OR 48 OR 49 OR 50  #52 exercise [Mesh Terms]  #53 exercise training [All Fields]  #54 exercise prescription [All Fields]  #55 continuous exercise training [All Fields]  #56 aerobic exercise [All Fields]  #57 aerobic training [All Fields]  #58 endurance training [All Fields]  #59 aerobic interval training [All Fields]  #60 interval exercise training [All Fields]  #61 high-intensity interval aerobic training [All Fields]  #62 resistance training [All Fields]  #63 strength [All Fields]  #64 52 OR 53 OR 54 OR 55 OR 56 OR 57 OR 58 OR 59 OR 60 OR 61 OR 62 OR 63  #65 51 OR 64  #66 arterial stiffness [All Fields]  #67 pulse wave velocity [All Fields]  #68 arterial destiffening [All Fields]  #69 aortic stiffness [All Fields]  #79 arterial health [All Fields]  #71 arterial elasticity [All Fields]  #72 vascular function [All Fields]  #73 66 OR 67 OR 68 OR 69 OR 70 OR 71 OR 72  #74 27 AND 32 AND 65 AND 73 |
