# Supplementary material for: Comparative effect of physical exercise versus statins on improving arterial stiffness in patients with high cardiometabolic risk: A network meta-analysis
Source: PLoS Med. 2021 Feb 16;18(2):e1003543. doi: 10.1371/journal.pmed.1003543 (PMC7924736; doi:10.1371/journal.pmed.1003543)
Supplement: S3 Table — (DOCX) [file pmed.1003543.s003.docx]

**S3 Table.** Pooled baseline characteristics from statins and physical exercise interventions

|  | **Age (years)** | | **BMI (kg/m^2^)** | | **SBP (mmHg)** | | **DBP (mmHg)** | | **Total Cholesterol (mmol/L)** | | **LDLc (mmol/L)** | | **HDLc (mmol/L)** | | **Triglycerides (mmol/L)** | | **HbA1c (%)** | | **cfPWv (m/s)** | |
| --- | --- | --- | --- | --- | --- | --- | --- | --- | --- | --- | --- | --- | --- | --- | --- | --- | --- | --- | --- | --- |
| **Statins interventions** | ***IG*** | ***CG*** | ***IG*** | ***CG*** | ***IG*** | ***CG*** | ***IG*** | ***CG*** | ***IG*** | ***CG*** | ***IG*** | ***CG*** | ***IG*** | ***CG*** | ***IG*** | ***CG*** | ***IG*** | ***CG*** | ***IG*** | ***CG*** |
| Davenport et al, 2015 (a) | 66.0±9.5 | - | 31.2±6.7 | - | 139.3±9.3 | - | 80.5±4.5 | - | 5.5±1.0 | - | 3.6±0.6 | - | NR | - | NR | - | 6.9±0.8 | - | 10.5±1.1 | - |
| Davenport et al, 2015 (b) | 65.5±10.5 |  | 32.4±4.7 |  | 139.5±5.0 |  | 77.3±7.75 |  | 5.6±1.2 |  | 3.4±0.8 |  | NR |  | NR |  | 6.8±0.8 |  | 10.3±1.5 |  |
| Fasset et al, 2019 | 62.3±16.3 | 64.8±15.0 | 28.4±5.8 | 28.7±5.3 | 140.5±13.0 | 147.0±20.9 | 76.5±10.6 | 78.8±7.8 | 6.9±1.8 | 7.6±2.2 | 4.0±1.4 | 4.9±1.6 | 1.7±1.4 | 1.7±0.6 | 1.1±0.7 | 1.2±0.8 | NR | NR | 8.5±2.2 | 8.0±1.3 |
| Grigoropoulou et al, 2019 | 60.0±8.0 | 59.0±9.0 | 30.3±4.7 | 30.7±5.9 | 140.0±17.0 | 140.0±17.0 | 77.0±9.0 | 78.0±8.0 | 6.1±0.7 | 5.9±1.0 | 4.0±0.7 | 3.9±0.6 | 1.3±0.2 | 1.2±0.2 | 1.6±0.8 | 1.7±0.9 | 6.7±0.7 | 7.0±0.7 | 11.2±2.7 | 10.7±2.4 |
| Kanaki et al, 2013 | 59.7±8.9 | 58.8±10.8 | 29.4±4.3 | 29.6±3.8 | 148±7.0 | 150.0±6.0 | 83.0±6.0 | 85.0±8.0 | 6.6±0.8 | 6.3±0.7 | 4.5±0.7 | 4.2±0.6 | 1.3±0.3 | 1.4±0.3 | 1.3±0.8 | 1.6±0.6 | 5.1±0.3 | 5.1±0.4 | 11.0±1.8 | 10.5±2.1 |
| Mitsiou et al, 2018 (a) | 52.8±8.2 | - | 29.2±4.3 | - | 122.4±7.4 | - | 83.6±6.8 | - | 6.4±0.7 | - | 4.3±0.7 | - | 1.4±0.2 | - | 1.6±0.6 | - | 5.3±0.3 | - | 8.4±1.2 | - |
| Mitsiou et al, 2018 (b) | 53.6±8.8 |  | 28.7±4.1 |  | 120.5±6.5 |  | 80.9±8.5 |  | 6.5±0.7 |  | 4.5±0.7 |  | 1.4±0.3 |  | 1.7±0.6 |  | 5.4±0.4 |  | 8.2±1.4 |  |
| Orr et al, 2009 | 53.0±7.2 | 55.0±9.0 | 31.9±3.2 | 31.1±2.7 | 129.0±14.4 | 127.0±12.0 | 74.0±7.2 | 75.0±6.0 | 5.5±0.5 | 5.9±0.8 | 3.9±0.6 | 4.2±0.6 | 1.1±0.3 | 1.1±0.4 | 1.4±0.6 | 1.4±0.6 | NR | NR | 11.0±1.3 | 12.4±2.7 |
| Pirro et al, 2006 | 56.0±17.0 | 58.0±14.0 | 26.1±5.6 | 25.8±4.3 | 129.0±10.0 | 131.0±11.0 | 75.0±14.0 | 74.0±10.0 | 6.7±0.7 | 6.7±0.9 | 4.7±0.5 | 4.6±0.7 | 1.3±0.2 | 1.3±0.3 | 1.6±0.7 | 1.6±0.8 | NR | NR | 9.5±1.9 | 9.2±2.1 |
| Raison et al, 2002 | 56.8±10.9 | 56.1±9.5 | 26.8±3.0 | 29.3±3.5 | 147.8±17.8 | 135.1±16.0 | 87.7±8.5 | 82.7±9.4 | 7.2±1.0 | 7.1±1.0 | 5.0±0.7 | 5.2±0.9 | 1.4±0.4 | 1.3±0.3 | NR | NR | NR | NR | 12.5±2.7 | 11.6±1.9 |
| Wang et al, 2011 | 64.2±7.8 | 65.7±8.2 | 24.8±4.9 | 25.3±3.6 | 161.1±8.2 | 164.5±9.3 | 89.1±7.9 | 87.2±7.2 | 5.8±0.6 | 5.8±0.7 | 4.6±0.4 | 4.7±0.5 | 1.1±0.1 | 1.1±0.3 | 2.1±0.3 | 2.1±0.3 | NR | NR | 14.4±2.7 | 14.3±3.1 |
| Yan et al, 2008 | 58.4±5.9 | 56.4±5.3 | 25.5±4.9 | 24.8±4.1 | 150.1±9.2 | 153.0±10.4 | 82.4±8.1 | 85.4±8.3 | 5.7±0.7 | 5.8±0.8 | 3.5±0.5 | 3.5±0.5 | 1.4±0.3 | 1.4±0.4 | 1.8±0.4 | 1.9±0.5 | NR | NR | 13.1±3.2 | 12.8±3.1 |
| Zhang et al, 2003 | 63.7±8.8 | 66.0±9.6 | 26.3±2.0 | 25.4±2.4 | 145.5±12.4 | 144.1±16.0 | 79.0±10.6 | 75.1±6.8 | NR | NR | NR | NR | NR | NR | NR | NR | NR | NR | 12.8±2.5 | 13.0±3.2 |
| **Pooled average** | **59.7±8.5** | **59.5±8.3** | **28.0±4.2** | **26.9±3.6** | **140.8±8.5** | **150.1±10.3** | **81.4±7.6** | **81.6±7.9** | **6.0±0.5** | **6.1±0.8** | **4.2±0.5** | **4.2±0.4** | **1.2±0.0** | **1.3±0.4** | **1.9±0.4** | **1.9±0.7** | **5.6±0.3** | **5.7±0.6** | **10.4±1.4** | **10.6±2.4** |
| **Physical exercise interventions** | | | | | | | | | | | | | | | | | | | | |
| Chrysohoou et al, 2015 | 63.0±8.8 | 56.0±11.0 | 28.9±4.2 | 31.3±7.0 | NR | NR | NR | NR | NR | NR | NR | NR | NR | NR | NR | NR | NR | NR | 9.5±2.5 | 8.8±1.3 |
| Dobrosielski et al, 2012 | 57.0±6.0 | 56.0±6.0 | 33.0±4.3 | 33.6±4.0 | 126.9±11.4 | 126.7±12.7 | 72.4±7.9 | 71.1±8.7 | NR | NR | NR | NR | NR | NR | NR | NR | 6.6±1.4 | 6.7±1.6 | 9.2±4.0 | 9.1±4.3 |
| Donley et al, 2014 | 46.0±4.0 | 44.0±3.0 | 38.0±2.0 | 34.0±2.0 | 125.0±5.0 | 128.0±4.0 | 80.0±2.0 | 81.0±2.0 | 4.9±0.3 | 4.9±0.3 | NR | NR | 1.3±0.1 | 1.0±0.1 | 1.3±0.2 | 1.8±0.3 | 5.6±0.1 | 5.6±0.1 | 7.9±0.6 | 7.4±0.4 |
| Guimaraes et al, 2010 (a) | 50.0±8.0 | 47.0±6.0 | 28.0±4.0 | 26.0±5.0 | 124.0±9.0 | 128.0±9.0 | 81.0±9.0 | 83.0±9.0 | NR | NR | NR | NR | NR | NR | NR | NR | NR | NR | 10.2±1.7 | 10.2±1.8 |
| Guimaraes et al, 2010 (b) | 45.0±9.0 |  | 29.0±5.0 |  | 125.0±9.0 |  | 81.0±5.0 |  | NR |  | NR |  | NR |  | NR |  | NR |  | 9.4±0.9 |  |
| Koh et al, 2010 (a) | 52.3±10.9 | 51.3±14.4 | 27.6±7.2 | 28.6±7.3 | 148.0±22.0 | 145.0±18.0 | 82.0±10.0 | 80.0±9.0 | NR | NR | NR | NR | NR | NR | NR | NR | NR | NR | 9.1±2.8 | 8.7±2.5 |
| Koh et al, 2010 (b) | 52.1±13.6 |  | 27.9±4.9 |  | 143.0±32.0 |  | 78.0±16.0 |  | NR |  | NR |  | NR |  | NR |  | NR |  | 9.7±3.2 |  |
| Loimaala et al, 2009 | 53.6±6.2 | 54.0±5.0 | 29.3±3.7 | 29.8±3.7 | 142.0±13.7 | 145.0±13.7 | NR | NR | 4.6±0.2 | 4.9±0.2 | 3.1±0.8 | 3.3±0.8 | 1.1±0.3 | 1.1±0.2 | 1.7±1.0 | 1.8±1.0 | 8.2±10.3 | 8.0±1.5 | 14.1±2.4 | 14.1±2.4 |
| Madden et al, 2013 | 68.5±4.5 | 70.0±4.2 | 30.9±5.0 | 28.6±4.2 | 148.0±20.0 | 140.0±15.6 | 82.0±15.0 | 82.0±10.4 | 4.9±1.0 | 5.0±1.5 | 2.7±1.0 | 2.6±1.0 | 1.5±0.5 | 1.6±1.0 | NR | NR | 6.8±1.5 | 6.6±1.0 | 13.4±3.5 | 12.0±3.3 |
| Mora-Rodriguez et al, 2017 | 53.5±8.9 | 53.5±8.9 | 32.3±3.1 | NR | 136.0±17.0 | 138.0±16.0 | 84.0±10.0 | 84.0±11.0 | 4.8±0.8 | 4.7±0.8 | 3.2±0.8 | 3.1±0.8 | 0.9±0.2 | 0.9±0.2 | 1.4±0.9 | 1.4±1.0 | NR | NR | 8.5±2.1 | 8.5±2.2 |
| Nualnim et al, 2012 | 58.0±9.8 | 61.0±8.7 | 29.0±4.9 | 32.0±4.4 | 131.0±14.7 | 129.0±17.4 | 76.0±9.8 | 76.0±8.7 | 5.2±1.0 | 5.5±1.1 | 3.3±1.0 | 3.5±1.1 | 1.5±0.5 | 1.4±0.4 | 1.4±0.7 | 1.5±0.9 | 4.7±0.5 | 4.5±0.4 | 9.1±1.0 | 9.4±0.2 |
| Rugolo et al, 2019 | 50.0±17.2 | 58.0±15.0 | 25.7±3.6 | 26.7±4.6 | NR | NR | NR | NR | 4.0±0.8 | 3.7±1.0 | 2.1±0.7 | 1.7±1.0 | 1.2±0.4 | 1.0±0.3 | 1.7±0.7 | 2.1±1.0 | NR | NR | 8.5±2.9 | 10.3±3.8 |
| Slivovskaja et al, 2019 | 53.9±6.4 | 52.0±7.7 | 30.8±4.0 | 31.0±3.5 | 132.6±13.0 | 132.4±14.9 | 81.5±9.3 | 82.1±10.7 | 6.4±1.4 | 5.8±1.3 | 4.2±1.2 | 3.7±1.1 | 1.2±0.3 | 1.1±0.3 | 2.4±2.8 | 2.0±0.9 | NR | NR | 8.5±1.4 | 8.0±1.1 |
| **Pooled average** | **54.2±7.2** | **54.8±9.0** | **30.1±3.2** | **30.4±2.5** | **133.0±6.9** | **134.2±6.7** | **79.7±4.0** | **79.7±5.1** | **5.0±0.6** | **4.9 ±0.3** | **3.1±0.7** | **3.0±0.6** | **1.2±0.2** | **1.1±0.5** | **1.6±0.3** | **1.8±0.2** | **5.6±0.3** | **5.6±0.3** | **9.7±1.3** | **9.6±0.8** |
