# Supplementary material for: Comparative effect of physical exercise versus statins on improving arterial stiffness in patients with high cardiometabolic risk: A network meta-analysis
Source: PLoS Med. 2021 Feb 16;18(2):e1003543. doi: 10.1371/journal.pmed.1003543 (PMC7924736; doi:10.1371/journal.pmed.1003543)
Supplement: S4 Table — (DOCX) [file pmed.1003543.s004.docx]

**S4 Table.** Quality grading of evidence

| **Certainty assessment** | | | | | | | **Number of patients** | | **Mean difference (95% CI)** | **Certainty** | **Importance** |
| --- | --- | --- | --- | --- | --- | --- | --- | --- | --- | --- | --- |
| **Number samples** | **Study design** | **Risk of bias** | **Inconsistency** | **Indirectness** | **Imprecision** | **Other considerations** | **Interventon** | **Control** |  |  |  |
| High-Statin dose compared to Control for Arterial stiffness | | | | | | | | | | | |
| 1 | RCT | Serious ^a^ | serious ^b^ | Not serious | Serious ^b^ | - Publication bias strongly suspected - very strong association - dose response gradient ^b^ | 16 | 10 | **-1.17 m/s**  (-3.5 to 1.16) | ⨁⨁⨁◯ MODERATE | NOT IMPORTANT |
| Moderate-Statin dose compared to Control for Arterial stiffness | | | | | | | | | | | |
| 7 | RCT | serious ^a^ | serious c | not serious | not serious | - strong association - dose response gradient | 225 | 215 | **-0.80 m/s**  (-1.59 to -0.01) | ⨁⨁⨁⨁ HIGH | CRITICAL |
| Low-Statin dose compared to Control for Arterial stiffness | | | | | | | | | | | |
| 1 | RCT | serious ^a^ | serious ^b^ | not serious | serious ^b^ | - publication bias strongly suspected - dose response gradient ^b^ | 45 | 42 | **-0.50 m/s** (-3.2 to 2.2) | ⨁◯◯◯ VERY LOW | NOT IMPORTANT |
| High-intensity Exercise compared to Control for Arterial stiffness | | | | | | | | | | | |
| 8 | RCT | serious ^a^ | not serious | not serious | not serious | - strong association - dose response gradient | 188 | 151 | **-0.56 m/s**  (-1.00 to -0.11) | ⨁⨁⨁⨁ HIGH | CRITICAL |
| Moderate-intensity Exercise compared to Control for Arterial stiffness | | | | | | | | | | | |
| 5 | RCT | serious ^a^ | not serious | not serious | not serious | - dose response gradient | 121 | 104 | **-0.27 m/s** (-1.00 to 0.46) | ⨁⨁⨁⨁ HIGH | IMPORTANT |
| High-Statin dose compared to Moderate-statin dose for Arterial stiffness | | | | | | | | | | | |
| 2 | RCT | serious ^a^ | not serious | not serious | not serious | - publication bias strongly suspected - dose response gradient ^b^ | 46 | 45 | **-0.11 m/s** (-0.8 to 0.59) | ⨁⨁⨁◯ MODERATE | NOT IMPORTANT |

**CI:** Confidence interval. a: Scored High in RoB2 tool; b: Few studies; c: Substantial heterogeneity
