# Supplementary material for: Comparative effect of physical exercise versus statins on improving arterial stiffness in patients with high cardiometabolic risk: A network meta-analysis
Source: PLoS Med. 2021 Feb 16;18(2):e1003543. doi: 10.1371/journal.pmed.1003543 (PMC7924736; doi:10.1371/journal.pmed.1003543)
Supplement: S5 Table — (DOCX) [file pmed.1003543.s005.docx]

**S5 Table.** Effectiveness ranking of stain doses and physical exercise intensities interventions.

|  | **Rank statistics** | | |  | **Probabilities** | |
| --- | --- | --- | --- | --- | --- | --- |
| **Interventions** | **Mean** | **Median** | **95% CI** |  | **Best** | **SUCRA** |
| Placebo | 4.2 | 5.5 | 1.0-6.0 |  | 0.00 | 0.14 |
| High-Statin dose | 3.2 | 3.5 | 1.0-5.0 |  | 0.35 | 0.74 |
| Moderate-Statin dose | 3.5 | 3.5 | 1.0-6.0 |  | 0.12 | 0.67 |
| Low-Statin dose | 4.0 | 4.5 | 2.0-6.0 |  | 0.35 | 0.49 |
| High-intensity Exercise | 2.8 | 3.0 | 1.0-5.0 |  | 0.12 | 0.60 |
| Moderate-intensity Exercise | 3.3 | 3.5 | 1.0-5.0 |  | 0.05 | 0.37 |
