# Supplementary material for: Comparative effect of physical exercise versus statins on improving arterial stiffness in patients with high cardiometabolic risk: A network meta-analysis
Source: PLoS Med. 2021 Feb 16;18(2):e1003543. doi: 10.1371/journal.pmed.1003543 (PMC7924736; doi:10.1371/journal.pmed.1003543)
Supplement: S6 Table — (DOCX) [file pmed.1003543.s006.docx]

**S6 Table.** Heterogeneity statistics for each pairwise comparison.

|  | **Q (df)** | **I^2^** | **τ^2^** | **p** |
| --- | --- | --- | --- | --- |
| High-Statin dose/ Placebo | 0.00 (0) | - | - | - |
| Moderate-Statin dose/ Placebo | 15.47 (6) | 61.2 | 0.650 | 0.017 |
| Low-Statin dose/ Placebo | 0.00 (0) | - | - | - |
| High-intensity Exercise/ Placebo | 0.26 (4) | 0.0 | 0.000 | 0.992 |
| Moderate-intensity Exercise/ Placebo | 1.40 (7) | 0.0 | 0.000 | 0.986 |
