# Supplementary figures and images for: Comparative effect of physical exercise versus statins on improving arterial stiffness in patients with high cardiometabolic risk: A network meta-analysis
Source: PLoS Med. 2021 Feb 16;18(2):e1003543. doi: 10.1371/journal.pmed.1003543 (PMC7924736; doi:10.1371/journal.pmed.1003543)

**S1 Fig.** Risk of bias for statin interventions.


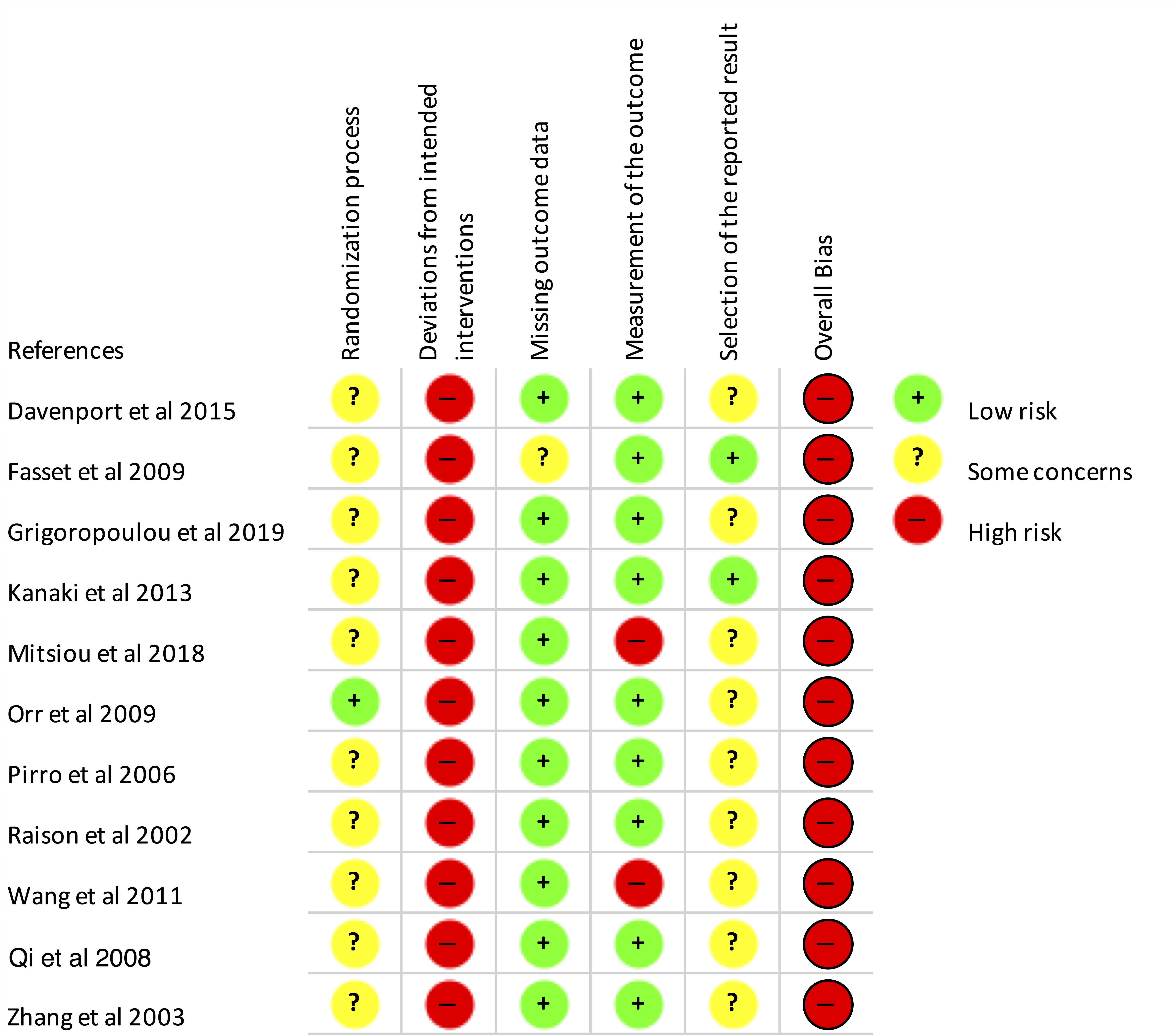

Supplement: S1 Fig — (DOCX) [file pmed.1003543.s007.docx]

**S2 Fig.** Risk of bias for physical exercise interventions


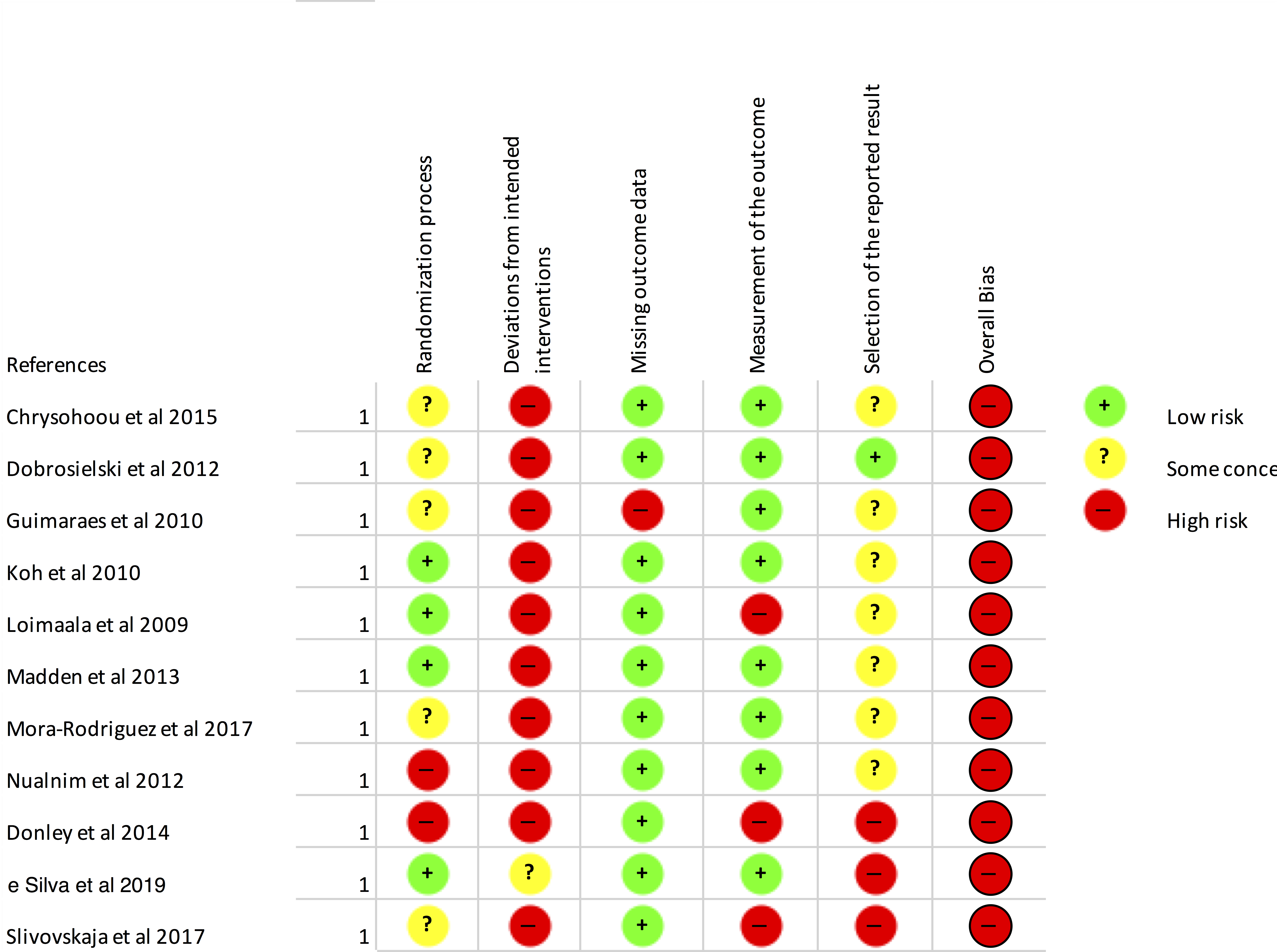

Supplement: S2 Fig — (DOCX) [file pmed.1003543.s008.docx]
