# Supplementary material for: Comparative effect of physical exercise versus statins on improving arterial stiffness in patients with high cardiometabolic risk: A network meta-analysis
Source: PLoS Med. 2021 Feb 16;18(2):e1003543. doi: 10.1371/journal.pmed.1003543 (PMC7924736; doi:10.1371/journal.pmed.1003543)
Supplement: S4 Fig — (DOCX) [file pmed.1003543.s010.docx]

**S4 Fig.** Funnel plot for comparison-specific pooled mean differences.
