# Supplementary material for: Comparative effect of physical exercise versus statins on improving arterial stiffness in patients with high cardiometabolic risk: A network meta-analysis
Source: PLoS Med. 2021 Feb 16;18(2):e1003543. doi: 10.1371/journal.pmed.1003543 (PMC7924736; doi:10.1371/journal.pmed.1003543)
Supplement: S1 Text — (DOCX) [file pmed.1003543.s011.docx]

**S1 Text. References of excluded studies (with reasons) from network meta-analysis.**

***Single-arm pre-post studies***

1. Canepa M, Artom N, Ameri P, et al. Short-term effect of rosuvastatin treatment on arterial stiffness in individuals with newly-diagnosed heterozygous familial hypercholesterolemia. Int J Cardiol 2018;255:215-20.
2. Castejon R, Castañeda A, Sollet A, et al. Short-term atorvastatin therapy improves arterial stiffness of middle-aged systemic lupus erythematosus patients with pathological pulse wave velocity. Lupus 2017;26(4):355-64.
3. Ikdahl E, Hisdal J, Rollefstad S, et al. Rosuvastatin improves endothelial function in patients with inflammatory joint diseases, longitudinal associations with atherosclerosis and arteriosclerosis: results from the RORA-AS statin intervention study. Arthritis Res Ther 2015;17(1):279.
4. Ikdahl E, Rollefstad S, Hisdal J, et al. Sustained improvement of arterial stiffness and blood pressure after long-term rosuvastatin treatment in patients with inflammatory joint diseases: results from the RORA-AS study. PloS one 2016;11(4):e0153440.
5. Jia X, Wei M, Fu X, et al. Intensive cholesterol-lowering therapy improves large artery elasticity in acute myocardial infarction patients. Heart Vessels 2009;24(5):340-6.
6. Kontopoulos AG, Athyros VG, Pehlivanidis AN, et al. Long-term treatment effect of atorvastatin on aortic stiffness in hypercholesterolaemic patients. Curr Med Res Opin 2003;19(1):22-7.
7. Muramatsu J, Kobayashi A, Hasegawa N, et al. Hemodynamic changes associated with reduction in total cholesterol by treatment with the HMG-CoA reductase inhibitor pravastatin. Atherosclerosis 1997;130(1-2):179-82.
8. Navarro-Muñoz M, Bonet J, Bayés B, et al. Atorvastatin treatment in the short term: does it induce renoprotection or vasculoprotection in renal transplantation?. Transplant Proc 2007;39(7):2259-63.
9. Ozaki K, Kubo T, Imaki R, et al. The anti-atherosclerotic effects of lipid lowering with atorvastatin in patients with hypercholesterolemia. J Atheroscler Thromb 2006;13(4):216-9.
10. Shinohara K, Shoji T, Kimoto E, et al. Effect of atorvastatin on regional arterial stiffness in patients with type 2 diabetes mellitus. J Atheroscler Thromb 2005;12(4):205-10.
11. Spaia S, Stavrati A, Panou E, et al. Effects of Atorvastatin on Aortic Pulse Wave Velocity (PWV) in Hemodialysis Patients: A Preliminary Study. Bantao J 2005;3(2):51.
12. Yamagishi T, Kato M, Koiwa Y, et al. Evaluation of plaque stabilization by fluvastatin with carotid intima-medial elasticity measured by a transcutaneous ultrasonic-based tissue characterization system. J Atheroscler Thromb 2009;16(5):662-73.

***Studies combining statins or physical exercise with other health interventions***

1. Hanžel J, Piletič Ž, Turk M, et al. A combination of low doses of fluvastatin and valsartan decreases arterial stiffness in patients after myocardial infarction: a pilot study. Curr Ther Res Clin Exp 2015;77:63-5.
2. Janić M, Lunder M, Prezelj M, et al. A combination of low-dose fluvastatin and valsartan decreases inflammation and oxidative stress in apparently healthy middle-aged males. J Cardiopulm Rehabil Prev 2014;34(3):208-12.
3. Janić M, Lunder M, Šabovič M. A low-dose combination of fluvastatin and valsartan: a new “drug” and a new approach for decreasing the arterial age. Biomed Res Int 2015;2015:235709.
4. Lunder M, Janić M, Jug B, et al. The effects of low-dose fluvastatin and valsartan combination on arterial function: a randomized clinical trial. Eur J Intern Med 2012;23(3):261-6.
5. Lunder M, Janić M, Savić V, et al. Very low-dose fluvastatin-valsartan combination decreases parameters of inflammation and oxidative stress in patients with type 1 diabetes mellitus. Diabetes Res Clin Pract 2017;127:181-6.
6. Mäki-Petäjä KM, Booth AD, Hall FC, et al. Ezetimibe and simvastatin reduce inflammation, disease activity, and aortic stiffness and improve endothelial function in rheumatoid arthritis. J Am Coll Cardiol 2007;50(9):852-8.
7. Savić V, Eržen B, Janić M, et al. Improvement of arterial wall characteristics by the low-dose fluvastatin and valsartan combination in type 1 diabetes mellitus patients. Diab Vasc Dis Res 2013;10(5):420-5.
8. Savić V, Janić M, Lunder M, et al. Long‑term improvement of arterial wall characteristics in patients with diabetes mellitus type 1 using cyclic, intermittent treatment with a low‑dose fluvastatin and valsartan combination. Exp Ther Med 2015;10(3):1207-11.
9. Svetek MB, Eržen B, Kanc K, et al. Impaired endothelial function and arterial stiffness in patients with type 2 diabetes—The effect of a very low-dose combination of fluvastatin and valsartan. J Diabetes Complications 2017;31(3):544-50.
10. Veselič MT, Eržen B, Hanžel J, et al. Improving Arterial Wall Characteristics in Patients After Myocardial Infarction with a Very Low Dose of Fluvastatin and Valsartan: A Proof-of-Concept Study. Med Sci Monit 2018;24:6892-9.
11. Turk MV, Žorž N, Eržen B, et al. Improvement of arterial wall phenotype in subjects at moderate cardiovascular risk induced by very low-dose fluvastatin/valsartan combination: a pilot study. Int Angiol 2018;37(5):356-64.

***Studies where the type and dose of statins or the intensity of physical exercise could not be estimated***

1. Du R, Zhao XQ, Cai J, et al. Changes in carotid plaque tissue composition in subjects who continued and discontinued statin therapy. J Clinical Lipidol 2016;10(3):587-93.
2. Okamoto T, Masuhara M, Ikuta K. Effects of eccentric and concentric resistance training on arterial stiffness. J Hum Hypertens 2006;20(5):348-54.
3. Okamoto T, Masuhara M, Ikuta K. Combined aerobic and resistance training and vascular function: effect of aerobic exercise before and after resistance training. J Appl Physiol 2007;103(5):1655-61.
4. Okamoto T, Masuhara M, Ikuta K. Effects of low-intensity resistance training with slow lifting and lowering on vascular function. J Hum Hypertens 2008;22(7): 509-11.
5. Okamoto T, Masuhara M, Ikuta K. Upper but not lower limb resistance training increases arterial stiffness in humans. Eur J Appl Physiol 2009;107(2):127-34.
6. Okamoto T, Masuhara M, Ikuta K. Effects of muscle contraction timing during resistance training on vascular function. J Hum Hypertens 2009;23(7):470-8.
7. Okamoto T, Masuhara M, Ikuta K. Effect of low-intensity resistance training on arterial function. Eur J Appl Physiol 2011;111(5):743-8.
8. Okamoto T, Masuhara M, Ikuta K. Low-intensity resistance training after high-intensity resistance training can prevent the increase of central arterial stiffness. Int J Sports Med 2013;34(5):385-90.
9. Okamoto T, Hashimoto Y, Kobayashi R. Effects of interval walking training compared to normal walking training on cognitive function and arterial function in older adults: a randomized controlled trial. Aging Clin Exp Res 2019;31(10):1451-9.

***Studies reporting arterial stiffness using other additional PWV measurements sites***

1. Choi KM, Han KA, Ahn HJ, et al. Effects of exercise on sRAGE levels and cardiometabolic risk factors in patients with type 2 diabetes: a randomized controlled trial. J Clin Endocrinol Metab 2012;97(10):3751-8.
2. Lee YS, Kim KS. The short-term effect of atorvastatin on flow-mediated vasodilation, pulse wave velocity and carotid intima-media thickness in patients with moderate cholesterolemia. Korean Circ J 2008;38(3):144-51.
3. Matsuo S, Nakamura Y, Yamada T, et al. Effect of statin therapy on arterial stiffness in patients with hyperlipidemia: Shiga Pravastatin Atherosclerosis Study (SHIPAS) Group. J Appl Res 2005;5(2):397.
4. Matsuo T, Iwade K, Hirata N, et al. Improvement of arterial stiffness by the antioxidant and anti-inflammatory effects of short-term statin therapy in patients with hypercholesterolemia. Heart Vessels, 2005;20(1):8-12.
5. Yokoyama H, Kawasaki M, Ito Y. Effects of fluvastatin on the carotid arterial media as assessed by integrated backscatter ultrasound compared with pulse-wave velocity. J Am Coll Cardiol 2005;46(11):2031-7.

***Studies no performed in cardio-metabolic disease risk patients***

1. Ciolac EG, Bocchi EA, Bortolotto LA, et al. Effects of high-intensity aerobic interval training vs. moderate exercise on hemodynamic, metabolic and neuro-humoral abnormalities of young normotensive women at high familial risk for hypertension. Hypertens Res 2010;33(8):836-43.
2. Cortez-Cooper MY, Anton MM, DeVan AE, et al. The effects of strength training on central arterial compliance in middle-aged and older adults. Eur J Cardiovasc Prev Rehabil 2008;15(2):149-55.
3. Goldberg MJ, Boutcher SH, Boutcher YN. The effect of 4 weeks of aerobic exercise on vascular and baroreflex function of young men with a family history of hypertension. J Hum Hypertens 2012;26(11):644-9.
4. Kim HK, Hwang CL, Yoo JK, et al. All-extremity exercise training improves arterial stiffness in older adults. Med Sci Sports Exerc 2017;49(7);1404-11.
5. Lunder M, Janić M, Habjan S, et al. Subtherapeutic, low-dose fluvastatin improves functional and morphological arterial wall properties in apparently healthy, middle-aged males–a pilot study. Atherosclerosis 2011;215(2):446-451.
6. Ramírez-Vélez R, Correa-Rodríguez M, Tordecilla-Sanders A, et al. Exercise and postprandial lipemia: effects on vascular health in inactive adults. Lipids Health Dis 2018;17(1):69.
7. Ramírez-Vélez R, Hernández-Quiñones PA, Tordecilla-Sanders A, et al. Effectiveness of HIIT compared to moderate continuous training in improving vascular parameters in inactive adults. Lipids Health Dis 2019;18(1):42.
8. Shenouda N, Gillen JB, Gibala MJ, et al. Changes in brachial artery endothelial function and resting diameter with moderate-intensity continuous but not sprint interval training in sedentary men. J Appl Physiol 2017;123(4):773-80.
9. Sugawara J, Akazawa N, Miyaki A, et al. Effect of endurance exercise training and curcumin intake on central arterial hemodynamics in postmenopausal women: pilot study. Am J Hypertens 2012;25(6):651-6.
