# Supplementary material for: Comparative effect of physical exercise versus statins on improving arterial stiffness in patients with high cardiometabolic risk: A network meta-analysis
Source: PLoS Med. 2021 Feb 16;18(2):e1003543. doi: 10.1371/journal.pmed.1003543 (PMC7924736; doi:10.1371/journal.pmed.1003543)
Supplement: Attachment — Submitted filename: caveroredondo.pdf [file pmed.1003543.s012.pdf]

This paper compares statins and exercise in various high risk groups using a network meta–analysis.

I was asked for a statistical report and I interpret that to include all aspects of the design and conduct of the study.

Points of detail

Page 7 Is the classification of exercise so obvious that we do not need to know who did it and whether it was double checked? I can see statins are easier to do.

Page 9 When I look at Figure 1 which is described here I find it difficult to determine which text goes with which edge. This is a common problem which could be solved I think by suppressing the dashed lines. I agree that once you have worked out that only solid lines have text it is obvious but it might be kinder to leave the dashed ones out.

Page 9 The authors do not in fact use values of I^2 and I agree that τ^2 is to be preferred but I wonder whether it is fair to compare heterogeneity between statins and exercise anyway. After all atorvastatin is the same the world over but the exact nature of endurance exercise must have differed between trials never mind the differences in adherence and trainer competence.

Page 10 The abstract says 19 studies, not 22. There do seem to be 22 in Figure 1 too.

Page Is this section supposed to be about transitivity? If so it needs labelling as such and also discussion later. Looking at Supplementary Table 3 it seems that the exercise trials took place in people with a more optimistic profile and so less improvement might have been possible.

Page 12 On page 10 the authors used both the phrase small study effects and publication bias but here just the latter. I think small study effects is superior unless you know the aetiology.

Page 14 I am not sure why the diverse clinical conditions of the participants is a limitation. If you want to generalise to real world practice is that not an advantage?

Supplementary Figure 1 I would have usually expected to find this in the main article as Figure 1. I think it is important.

Supplementary Figure 5 The studies do seem to be very closely clustered round the common estimates. Is there any obvious explanation for this apart from chance?

Supplementary references This is a very desirable inclusiom.

There are one or two typos (ardiovascular and immprove on p4 for instance) but satatin in Figure 2 stands out. Incidentally why control here but placebo elsewhere?

Point of more substance

Observing the field of cardiovascular risk from afar I notice even in the popular media that statins have raised some controversy. To put it mildly. I wonder what their proponents will make of the authors' decision to focus on medium dose statins and high dose exercise despite the SUCRA showing high dose statins to be superior. After all looking at Table 3 we see that the central estimates for high and medium dose versus placebo are effectively equal so it could be argued that this is just a matter of precision that one confidence interval includes the null and the other does not. Some more argument and justification is needed here I think. I am not saying there is anything wrong with the authors' conclusions, given the evidence here if I were in a high risk group I would prefer to be prescribed exercise than high dose statins. Others might not be so easily convinced.

Summary

Mostly details and requests for clarification.

Michael Dewey
