# Supplementary material for: Comparative effect of physical exercise versus statins on improving arterial stiffness in patients with high cardiometabolic risk: A network meta-analysis
Source: PLoS Med. 2021 Feb 16;18(2):e1003543. doi: 10.1371/journal.pmed.1003543 (PMC7924736; doi:10.1371/journal.pmed.1003543)
Supplement: Attachment — Submitted filename: Response to reviewers.docx [file pmed.1003543.s013.docx]

Dear Emma Veitch, PhD,

PLOS Medicine

Enclosed you will find a revision of our manuscript**: Comparative effect of physical exercise versus statins on improving arterial stiffness in patients with high cardiometabolic risk: a network meta-analysis.** Manuscript ID PMEDICINE-D-20-03097R1.

We would like to thank you for giving us the opportunity to revise and improve our manuscript; we also thank the reviewers for their thoughtful and constructive comments.

We have considered all of the suggestions and have incorporated them into the revised manuscript. Changes to the original manuscript are highlighted in red, and we believe our manuscript is stronger as a result of these modifications. An itemized point-by-point response to the editor and reviewers’ comments is presented below.

Celia Álvarez-Bueno

Universidad de Castilla-La Mancha

Edificio Melchor Cano, Centro de Estudios Socio-Sanitarios

Santa Teresa Jornet s/n, 16071 Cuenca, Spain.

E-mail: celia.alvarezbueno@uclm.es

Telephone: + (34) 969179100 ext. 4659

**Requests from the editors:**

*In the Abstract (ideally Methods and Findings section), please include a brief note summarising any key limitation(s) of the study's methodology.

**Authors:**

Thank you for the editors' comment. As suggested, we have included a brief note summarising the main limitation of the study's methodology in the Abstract section as follows:

“ **Methods and findings:**

[…]. For standard pairwise meta-analysis and a network meta-analysis, high-intensity exercise versus control (mean difference (MD) -0.82; 95%CI: -1.48, -0.15; and -0.80; 95%CrI: -1.55, -0.06, respectively) and moderate-statin dose versus control (MD -0.80, 95%CrI: -1.59, -0.01; and -0.74, 95%CrI: -1.33, -0.15, respectively) showed significant mean differences. *The magnitude of the effect of the exercise interventions could be underestimated due to regression toward the mean bias, beause the baseline cardiometabolic risk profile of subjects in the physical exercise intervention trials was healthier than those in the statins ones, consequently more modest improvements in physical exercise interventions compared to statins interventions can be expected.*”

*The main Limitations section of the paper seems to omit mention of a key point, that is the focus specifically in this network meta on a surrogate outcome measure - some discussion of this could be added.

**Authors:**

Editors' comment seems judicious. As suggested, a limitation on the surrogate outcome has been included and discussed as follows:

“[…]. *Finally, our results are based on the analysis of the effect of statins and exercise on reducing PWV, and although consistent evidence support that PWV is a good predictor of CVD, it is indeed a surrogate measure of the appropriate main outcomes for these interventions, which are CVD events, CVD mortality and all cause mortality, with all the clinical and epidemiological implications that this fact entails.*”

*The authors have used the PRISMA guideline (specifically the one focussed on network meta-analyses) to help with reporting, and this is valuable, but a "flowchart" of study eligibility/inclusion seems to have been omitted - please see the generic PRISMA statement (https://www.equator-network.org/reporting-guidelines/prisma/) for a template Word figure for the flowchart and that could be filled out and included as a figure with the revised paper.

**Authors:**

Thank you for the editors' comment. As suggested, flowchart has been included in the main manuscript as Figure 1.

**Comments from the reviewers:**

**Reviewer 1**

This paper compares statins and exercise in various high risk groups using a network meta–analysis.

I was asked for a statistical report and I interpret that to include all aspects of the design and conduct of the study.

Points of detail

Page 7 Is the classification of exercise so obvious that we do not need to know who did it and whether it was double checked? I can see statins are easier to do.

**Authors:**

The reviewer's comment seems judicious. As in the search, data extraction and risk of bias assessment of the studies, the categorization of the interventions was done by two researchers in order to have a double check. As suggested, the following sentence has been included in the ‘Categorization of the interventions’ available evidence’ section:

“[…]Furthermore, physical exercise interventions, considered as a subset of structured and repetitive physical activity, were classified by intensity as high, moderate or light following the American College of Sport Medicine guidelines for prescribing exercise.^27^ *This classification of statin doses and exercise intensity was performed by two researchers (IC-R and CA-B) independently.*”

Page 9 When I look at Figure 1 which is described here I find it difficult to determine which text goes with which edge. This is a common problem which could be solved I think by suppressing the dashed lines. I agree that once you have worked out that only solid lines have text it is obvious but it might be kinder to leave the dashed ones out.

**Authors:**

Thank you for the reviewer's comments. As suggested, dashed lines have been suppressed from Figure 2 (formerly Figure 1).

Page 9 The authors do not in fact use values of I2 and I agree that τ2 is to be preferred but I wonder whether it is fair to compare heterogeneity between statins and exercise anyway. After all atorvastatin is the same the world over but the exact nature of endurance exercise must have differed between trials never mind the differences in adherence and trainer competence.

**Authors:**

Thank you for the reviewer’s thoughtful comment. As suggested, the heterogeneity analysis was done separately for each statin dose and for each exercise intensity. We would like to apologize for the misunderstanding because it was not clear from the statistical methods section. This section has been reworded to make it clearer as follows:

“[…] the statistical heterogeneity was examined by calculating the I^2^ statistic, *separetly for each statin doses and for each exercise intensity*, which ranges from 0% to 100%. […]”

Page 10 The abstract says 19 studies, not 22. There do seem to be 22 in Figure 1 too.

**Authors:**

Thank you for the reviewer's comments. As suggested, the number of studies has been modified in the abstract and in Figure 2 (formerly Figure 1).

Page Is this section supposed to be about transitivity? If so it needs labelling as such and also discussion later. Looking at Supplementary Table 3 it seems that the exercise trials took place in people with a more optimistic profile and so less improvement might have been possible.

**Authors:**

Thank you for the reviewer's comments. As suggested, we have labelled the transitivity section in results section. Additionally we have included the following sentence in the discussion section:

“[…]*. Fourth, the transitivity assessment showed a lower risk cardiometabolic profile at baseline in the subjects in physical exercise interventions trials than those in statins interventions, consequently more modest improvements in physical exercise interventions compared to statins interventions can be expected* […]”

Page 12 On page 10 the authors used both the phrase small study effects and publication bias but here just the latter. I think small study effects is superior unless you know the aetiology.

**Authors:**

The reviewer's comment seems judicious. As suggested, and following Sterne et al., small study effect is a possible, probably the most well-known, reason of publication bias. Thus, we have replaced the term publication bias by small study effect.

Page 14 I am not sure why the diverse clinical conditions of the participants is a limitation. If you want to generalise to real world practice is that not an advantage?

**Authors:**

Thank you for the reviewer's comments. As susgested, we have included a sentence pointing out that this diversity is an advantage as follows:

“[…]. Fifth, a high heterogeneity in the characteristics of population included in the studies such as clinical (including patients with hypertension, obesity, diabetes mellitus, chronic kidney disease or heart failure), or cardiometametabolic risk profile; *certainly, this heterogeneity in cardiovascular risk status affects the statistical procedures of the study and their estimates must be cautiously examined, although it also makes our findings generalizable to real clinical practice*. […]”

Supplementary Figure 1 I would have usually expected to find this in the main article as Figure 1. I think it is important.

**Authors:**

Thank you for the reviewer's comments. As susgested, we have included Supplementary Figure 1 as Figure 1 in the main article.

Supplementary Figure 5 The studies do seem to be very closely clustered round the common estimates. Is there any obvious explanation for this apart from chance?

**Authors:**

Thank you for the reviewer’s thoughtful comment. As suggested, we have included a sentence explaining this phenomenon as follows:

“[…]. Seventh, there was evidence of small study effect on the funnel plot asymmetry (Supplementary Figure 4) and Egger test, for high-intensity exercise versus control comparison, produced mainly by the scarcity of studies and their small sample sizes. *Furthermore, the funnel plot asymmetry showed that the studies appear to be highly clustered round common estimates for each type of intervention, this phenomenon can be explained by the similarity of sample sizes, but in addition to the reliability of the direction and the power of the effect for each type of intervention*. […]”

Supplementary references This is a very desirable inclusiom.

**Authors:**

We really appreciate the reviewer's comment.

There are one or two typos (ardiovascular and immprove on p4 for instance) but satatin in Figure 2 stands out. Incidentally why control here but placebo elsewhere?

**Authors:**

Thank you for the reviewer's comments. An extensive review has been performed in order to detect this typo mistakes. Additionally, we have replaced control by placebo in Figure 3 (formerly Figure 2).

Point of more substance

Observing the field of cardiovascular risk from afar I notice even in the pop- ular media that statins have raised some controversy. To put it mildly. I wonder what their proponents will make of the authors’ decision to focus on medium dose statins and high dose exercise despite the SUCRA showing high dose statins to be superior. After all looking at Table 3 we see that the central estimates for high and medium dose versus placebo are effectively equal so it could be argued that this is just a matter of precision that one confidence interval includes the null and the other does not. Some more argument and justification is needed here I think. I am not saying there is anything wrong with the authors’ conclusions, given the evidence here if I were in a high risk group I would prefer to be prescribed exercise than high dose statins. Others might not be so easily convinced.

**Authors:**

Thank you for the reviewer’s thoughtful comment. As suggested, we have included the following sentence in the discussion section:

“[…]. Our analysis covered 1312 cardio-metabolic disease/risk patients (592 for statins and 720 for physical exercise), but only a limited number of studies examined the effect of high- and low-statin doses as well as for light- intensity exercise. Even so, with the studies included, a dose response trend can be assumed both for statins and exercise. *This is evident from the SUCRA findings that showed that the high-statin dose may be the best treatment, but the limited number of studies may not find a statistically significant effect, but it should be highlighted that when choosing the safest treatment, increasing the dose of statins has been associated with pain and muscle damage, liver injury or increased risk of type 2 diabetes.* ^58,59^ Conversely, exercise have demonstrated positive benefits for most prevalent non-communicable diseases.^60^”

Summary

Mostly details and requests for clarification.

**Authors:**

Thank you for the reviewer's contributions and comments on the statistical methods.

**Reviewer 2**

The Authors of this paper carried out a network meta-analysis on the effect of different statins doses and exercise intensities on arterial stiffness (expressed as carotid-femoral pulse wave velocity), in high cardio-metabolic risk patients. Nineteen studies were included in the analyses, including 1151 patients.

The main results showed in addition to a benefit from statins for reducing cardiovascular risk, also a beneficial effects of high-intensity exercise on arterial stiffness.

This is an interesting paper, however there are some concerns.

1. The results may be affected by some limitations: a) the high overall risk of bias in all included studies; b) the inclusion of non-randomized controlled trials; c) the limited number of samples included in the evaluation of some interventions (high-statin dose vs control n=1, low-statin dose vs control n=1), that also affected the GRADE evaluation; d) the statistical difference in baseline cardio-metabolic risk profile between physical exercise and statin intervention group; e) a high heterogeneity in the characteristics of the studies (e.g. cardio-metabolic risk profile of the populations, arterial stiffness assessment).

**Authors:**

The reviewer's comment seems judicious. As suggested, we have modified and included this limitations in discussion section as follows:

“*The results of this network meta-nalysis may be affected by some limitations that should be acknowledged. First, the high overall risk of bias in all included studies, mainly due to all trials lacked information regarding deviations from intended interventions domain from the RoB2; however, based on the others quality domains of the RoB2, the methodological quality of included trials was generally satisfactory. Second, the inclusion of non-randomized experimental studies and controlled pre–post studies, although the exclusion of the non-randomized experimental studies in the sensitivity analysis was only reflected in small modifications of the effect in both moderate-statin dose versus control and high-intensity exercise versus control. Third, the limited number of samples included in the evaluation of some interventions (high-statin dose versus control n=1 and low-statin dose versus control n=1), which influenced the GRADE evaluation. Fourth, the transitivity assessment showed a lower risk cardiometabolic profile at baseline in the subjects in physical exercise interventions trials than those in statins interventions, consequently more modest improvements in physical exercise interventions compared to statins interventions can be expected. Fifth, a high heterogeneity in the characteristics of population included in the studies such as clinical (including patients with hypertension, obesity, diabetes mellitus, chronic kidney disease or heart failure), or cardiometametabolic risk profile; certainly, this heterogeneity in cardiovascular risk status affects the statistical procedures of the study and their estimates must be cautiously examined, although it also makes our findings generalizable to real clinical practice. Sixth, an additional source of heterogeneity is that the devices used for measuring cfPWv differ largely, since while some used a tonometric method, other were oscillometric devices, but the robustness of our estimates was not modified after removing studies including oscillometric devices in the sensitivy analyses. Seventh, there was evidence of small study effect on the funnel plot asymmetry (Supplementary Figure 4) and Egger test, for high-intensity exercise versus control comparison, produced mainly by the scarcity of studies and their small sample sizes. Furthermore, the funnel plot asymmetry showed that the studies appear to be highly clustered round common estimates for each type of intervention, this phenomenon can be explained by the similarity of sample sizes, but in addition to the reliability of the direction and the power of the effect for each type of intervention. Finally, our results are based on the analysis of the effect of statins and exercise on reducing PWV, and although consistent evidence support that PWV is a good predictor of CVD, it is indeed a surrogate measure of the appropriate main outcomes for these interventions, which are CVD events, CVD mortality and all cause mortality, with all the clinical and epidemiological implications that this fact entails.*”

2. The visual inspection of the funnel plot pointed out a slight asymmetrical distribution. Moreover, the result of Egger's test was not reported in the text.

**Authors:**

Thank you for the reviewer’s thoughtful comment. First, we apologize because re-analysing data we have found a mistake, and there is evidence of publication bias in high-intensity exercise versus control comparison. Therefore, apart from the inclusion of p-values of Egger test, we have amended the ‘Sensitivity analysis, heterogeneity and small study effect’ section, as well as the discussion section as follows:

In Sensitivity analysis, heterogeneity and small study effect section:

“.[…]. Finally, *there was evidence of small study effect in funnel plot asymmetry and Egger test for high-intensity exercise versus control (p= 0.035), but not for all other comparisons: moderate-statin dose versus control (p= 0.294), moderate-statin dose versus high-statin dose (p= 0.686) and moderate-intensity exercise versus control (p= 0.557) (Supplementary Figure 4).*”

In discussion section:

“[…]. *Seventh, there was evidence of small study effect on the funnel plot asymmetry (Supplementary Figure 4) and Egger test, for high-intensity exercise versus control comparison, produced mainly by the scarcity of studies and their small sample sizes. Furthermore, the funnel plot asymmetry showed that the studies appear to be highly clustered round common estimates for each type of intervention, this phenomenon can be explained by the similarity of sample sizes, but in addition to the reliability of the direction and the power of the effect for each type of intervention.* […]”

3. Please consider another reference for the effect of statins on arterial stiffness, expressed only as carotid-femoral pulse wave velocity (Clin Exp Hypertens. 2018;40(7):601-608. doi: 10.1080/10641963.2017.1411498), because Upala et al (ref 11) combined the results of different expression of arterial stiffness.

**Authors:**

Thank you for the reviewer's comments. As suggested we have included the recommended reference.

“*D’elia L, La Fata E, Iannuzzi A, Rubba PO. Effect of statin therapy on pulse wave velocity: A meta-analysis of randomized controlled trials. Clinical and Experimental Hypertension 2018;40(7):601-608*.”

4. Please check typo-errors at page 4,9, and Figure 2

**Authors:**

Done. In addition, an extensive review has been performed in order to detect this typo mistakes. Thank you.

5. Please re-check the references (e.g. reference 12, it should be Upala et al)

**Authors:**

Thank you for the reviewer's comment. As suggested, we have re-checked the references.

6. The different number of samples/studies included reported in the abstract and manuscript may generate confusion

**Authors:**

Thank you for the reviewer's comments. As suggested, the number of studies has been modified in the abstract.

**Reviewer 3**

Although many patients could benefit from statins to reduce CVD risk, our results support that, considering the beneficial effects of high-intensity exercise on arterial stiffness, it would be worthwhile to refocus our attention on this type of exercise as an effective tool for prevention of CVD.

A well written paper with practical implications.

Any issues with the original submission were resolved in the revision in a satisfactory way.

**Authors:**

We really appreciate the reviewer's comments.
