# Supplementary material for: Comparative effect of physical exercise versus statins on improving arterial stiffness in patients with high cardiometabolic risk: A network meta-analysis
Source: PLoS Med. 2021 Feb 16;18(2):e1003543. doi: 10.1371/journal.pmed.1003543 (PMC7924736; doi:10.1371/journal.pmed.1003543)
Supplement: Attachment — Submitted filename: Response to Reviewers .docx [file pmed.1003543.s014.docx]

Dear Artur Arikainen, PhD,	January 2021

Associate Editor

PLOS Medicine

Enclosed you will find a revision of our manuscript**: Comparative effect of physical exercise versus statins on improving arterial stiffness in patients with high cardiometabolic risk: a network meta-analysis.** Manuscript ID PMEDICINE-D-20-03097R2.

We would like to thank you for giving us the opportunity to revise and improve our manuscript; we also thank the editors for their thoughtful and constructive comments.

We have considered all of the suggestions and incorporated them into the revised manuscript. Changes in the original manuscript are highlighted in red, and we believe our manuscript is stronger as a result of these modifications. An itemized point-by-point response to the editors’ comments is presented below.

Celia Álvarez-Bueno

Universidad de Castilla-La Mancha

Edificio Melchor Cano, Centro de Estudios Socio-Sanitarios

Santa Teresa Jornet s/n, 16071 Cuenca, Spain.

E-mail: celia.alvarezbueno@uclm.es

Telephone: + (34) 969179100 ext. 4659

**Requests from Editors:**

1. Financial Disclosure: Please add “The funders had no role in study design, data collection and analysis, decision to publish, or preparation of the manuscript.”; or explain otherwise.

**Authors:** Done. Thank you.

2. Data Availability Statement (DAS): If the data are owned by a third party but freely available upon request, please note this and state the owner of the data set and contact information for data requests (web or email address). Note that a study author cannot be the contact person for the data. If the data are not freely available, please describe briefly the ethical, legal, or contractual restriction that prevents you from sharing it. Please also include an appropriate contact (web or email address) for inquiries (again, this cannot be a study author).

**Authors:** As requested, we have included the following information.

“***Data Availability***

*All relevant data are within the manuscript and its Supporting Information files*.”

3. Please include line numbers in the margin throughout.

**Authors:** Done. Thank you.

4. Abstract:

a. Please include search dates, and databases searched.

**Authors:** Thank you for the editors’ comment. As suggested, we have included in the abstract the following information:

“**Methods and findings:** *We systematically searched MEDLINE, EMBASE, SPORTDiscus, Cochrane Central Register of Controlled Trials, Cochrane Database of Systematic Reviews and Web of Science databases from their inception to February 2020; for unpublished trials, we also searched clinicaltrials.gov.* […]”

b. Please include a summary of included study types (RCTs, non-randomized cohorts, etc.), and a summary of study location by global region/country.

**Authors**: Thank you for the editors’ comment. As suggested, we have included in the abstract the following information:

“[…]. Twenty-two studies were included in the analyses *(18 RCTs and four non-randomized experimental studies)*, including 1307 patients with high cardiometabolic risk *from Asia, Oceania, Europe, North America South America*. […]

c. Please include the name of the quality/bias assessment method, and a summary of included study quality.

**Authors:** Thank you for the editors’ comment. As suggested, we have included in the abstract the following information:

“[…]. *For methodological quality assessment, Cochrane Collaboration’s tool for assessing risk of bias (RoB2) was used.* […].”

“[…]. *The overall risk of bias assessed with RoB2 was high in all included studies*. […]”

d. Please include p values alongside 95% CIs/CrIs for comparisons.

**Authors:** Thank you for the editors’ comment. As suggested, we have included p values.

e. Please mention the results of the sensitivity analysis (removal of non-randomized studies).

**Authors:** Thank you for the editors’ comment. As suggested, we have included in the abstract the following information:

“[…]. *When non-randomized experimental studies were excluded, the effect on high-intensity exercise versus control and moderate-statin dose versus was slightly modified.* […]”

f. Please clarify where in the main Results/Tables the following data values can be verified (we were unable to locate all of these): “For standard pairwise meta-analysis and a network meta-analysis, high-intensity exercise versus control (mean difference (MD) -0.82; 95%CI: -1.48, -0.15; and -0.80; 95%CrI: -1.55, -0.06, respectively) and moderate-statin dose versus control (MD -0.80, 95%CrI: -1.59, -0.01; and -0.74, 95%CrI: -1.33, -0.15, respectively) showed significant mean differences.”

**Authors:** Thank you for the editors’ comment. We would like to apologize for the misunderstanding, as the information in the Arterial stiffness section of results was modified in a previous revision, but we did not do it in the abstract. We have unified the information in both sections.

g. At the end of the ‘Methods and findings’ subsection, please more clearly state: “Limitations of this study were…”; and mention other major issues, such as small study sizes, heterogeneous patient groups, focus on a proxy endpoint, and high risk of bias.

**Authors:** Thank you for the editors’ comment. As suggested, we have included in the abstract the following information:

“[…]. *The main limitation of this study was* that the magnitude of the effect of the exercise interventions could be underestimated due to regression toward the mean bias, because the baseline cardiometabolic risk profile of subjects in the physical exercise intervention trials was healthier than those in the statins ones, consequently more modest improvements in physical exercise interventions compared to statins interventions can be expected. *Additionally, we might consider as limitations the small study sizes; the heterogeneous patient groups; the focus on a proxy endpoint (PWV), and the high risk of bias.*”

h. Conclusions: Please begin with: “In this network meta-analysis, we found that…”

**Authors:** Done. Thank you.

5. Author Summary: Under "What do these findings mean", please mention that choices of interventions will need to be based on the needs and preferences of individual patients (i.e., not everyone can do high-intensity exercise, and statins have their drawbacks too).

**Authors:** The editor’s comment seems judicious. As suggested, we have included the following statement:

“*- However, the choice of interventions should be based on the needs and preferences of individual patients.*”

6. Please use the "Vancouver" style for reference formatting, and see our website for other reference guidelines https://journals.plos.org/plosmedicine/s/submission-guidelines#loc-references. Citations should be in square brackets, before punctuation, and not superscript, eg. “…[1,2].”

**Authors:** Done. Thank you.

7. Methods:

a. Please include the exact search date (including day).

**Authors:** Done. Thank you.

b. Please mention that separate ethical approval was not required for your study.

**Authors:** Thank you for the editors’ comment. As suggested, we have included the following statement in Methods section:

“[…]. *Since in this study the researchers did not collect primary data for the systematic review and network meta-analysis, ethical approval was not required*.”

8. Results: Please include p values alongside 95% CIs/CrIs for all comparisons.

**Authors:** Thank you for the editors’ comment. As suggested, we have included p values.

9. Please remove the Contributors, Funding, and Disclosures sections on p. 16-17 – these details are taken from the online submission from.

**Authors:** Done. Thank you.

10. Please provide access details (eg. volume/issue/pages, DOI or URL) for references 9, 22, 26.

**Authors:** Done. Thank you.

11. Please upload each item of Supporting Information as a separate file.

**Authors:** Done. Thank you.

12. When completing the PRISMA checklist, please use section and paragraph numbers, rather than page numbers.

**Authors:** Done. Thank you.

**Comments from Reviewers:**

Reviewer #1: The authors have addressed all my points

Michael Dewey

**Authors:** Thank you for the reviewer’s comment. We greatly appreciate the involvement of the reviewer in the review of the manuscript.

Reviewer #2: The paper was improved. No further comments.

**Authors:** Thank you for the reviewer’s comment. We greatly appreciate the involvement of the reviewer in the review of the manuscript.
