# Supplementary material for: Comparative effect of physical exercise versus statins on improving arterial stiffness in patients with high cardiometabolic risk: A network meta-analysis
Source: PLoS Med. 2021 Feb 16;18(2):e1003543. doi: 10.1371/journal.pmed.1003543 (PMC7924736; doi:10.1371/journal.pmed.1003543)
Supplement: Attachment — Submitted filename: Response to Reviewers.docx [file pmed.1003543.s015.docx]

Dear Artur Arikainen, PhD,	January 2021

Associate Editor

PLOS Medicine

Enclosed you will find a revision of our manuscript**: Comparative effect of physical exercise versus statins on improving arterial stiffness in patients with high cardiometabolic risk: a network meta-analysis.** Manuscript ID PMEDICINE-D-20-03097R3.

We have considered all of the suggestions and incorporated them into the revised manuscript. Changes in the original manuscript are highlighted in red, and we believe our manuscript is stronger as a result of these modifications. An itemized point-by-point response to the editors’ comments is presented below.

Celia Álvarez-Bueno

Universidad de Castilla-La Mancha

Edificio Melchor Cano, Centro de Estudios Socio-Sanitarios

Santa Teresa Jornet s/n, 16071 Cuenca, Spain.

E-mail: celia.alvarezbueno@uclm.es

Telephone: + (34) 969179100 ext. 4659

**Requests from Editors:**

1. Abstract:

a. Line 47: Please give numbers of studies included from each region.

**Authors:** Done. Thank you

b. Line 55: Delete duplicate “The main”

**Authors:** Done. Thank you

2. Some final additional comments from the Academic Editor for consideration (apologies that these were not included in our previous email):

a. Methods/interpretation: The authors should acknowledge and discuss that network meta-analysis (in particular when comparisons are indirect) is by many considered as prone to the same issues that non-randomised comparisons are subject to. So, all indirect comparisons between PA and statins are potentially biased and should be carefully interpreted.

**Authors:** The editor’s comment seems judicious. As suggested, we have included the following statement in limitations section:

“[…]. *Eighth, it should be acknowledged that because in this network meta-analysis, comparisons between physical exercise and statins are indirect, the same biases may occur as in non-random comparisons, and the results should be carefully interpreted.* […]”

b. Intro/discussion: I recommend that the authors tone down the PA vs statin debate and recommendations. It is not really one or the other but the emphasis should be on both (and this paper arguing that we should put more emphasis on PA - but not at the cost of drug treatment).

**Authors:** We would like to thank the editor’s comment. As suggested, we have toned down the PA vs statin debate and recommendations. We have modified some sentences and words from introduction as follows:

“[…]. The comparative analysis of the effect of several doses of statins and different intensities of physical exercise on arterial stiffness *could shed light for clinicians on the effectiveness of both methods in preventing CVD* and be used to inform shared decision making between doctors and patients”

“There is a worldwide concern as to whether the undeniable cardioprotective benefits of the statins *have masked* the importance of the exercise in the prevention of CVD. […]”

“However, there is a lack of clinical trials comparing *the effect of statins and physical exercise* on most cardiovascular disease risk outcomes such as arterial stiffness, and standard meta-analyses are unable to resolve which treatment is more effective. […]”

“[…]. *Although many appropriately selected patients could benefit from the effects of statins on arterial stiffness, our results support that, considering the beneficial effects of high-intensity exercise on arterial stiffness, the choice of this intervention should be based on the needs and preferences of each patient*.”

c. Discussion: Following from the point above, one key limitation of the work is that focuses on one surrogate outcome but then draws far-reaching conclusions on clinical and public health decision making. This is stretching things too far and should be emphasised.

**Authors:** The editor’s comment seems judicious. As suggested, we have modified conclusions on clinical and public decision making as follows:

“[…]. *Although many appropriately selected patients could benefit from the effects of statins on arterial stiffness, our results support that, considering the beneficial effects of high-intensity exercise on arterial stiffness, the choice of this intervention should be based on the needs and preferences of each patient*.”

3. Line 369: Typo in “meta-analysis”.

**Authors:** Corrected. Thank you

4. Lines 414-420: Please remove the Data Availability and financial Disclosures sections – these details are taken from the online submission form.

**Authors:** Done. Thank you.

5. Reference 63: Please remove italic formatting here.

**Authors:** Done. Thank you.

6. When completing the PRISMA checklist, please use section and paragraph numbers, rather than page or line numbers – page and line numbers will change in the final published document.

**Authors:** Done. Thank you
